# Efficacy and tolerability of chemotherapy in Chinese patients with AIDS-related Burkitt lymphoma and diffuse large B-cell lymphoma: An observational study

**DOI:** 10.1038/s41598-017-02086-4

**Published:** 2017-05-15

**Authors:** Jiang Xiao, Shuxu Du, Guorui Dai, Guiju Gao, Di Yang, Hongxin Zhao

**Affiliations:** 1 Clinical and Research Center of Infectious Diseases, Beijing Ditan Hospital, Capital Medical University, Beijing, 100015 China; 2 Beijing Shijitan Hospital, Capital Medical University, Beijing, 100020 China

## Abstract

We evaluated the efficacy and tolerability of chemotherapy in HIV-infected patients with diffuse large B-cell lymphoma (DLBCL) receiving CHOP ± R (*n* = 17) or Burkitt lymphoma (BL) receiving CODOX-M/IVAC ± R (*n* = 15). The study was conducted in Beijing Ditan Hospital from January 2009 to August 2015. The following grade 4 adverse effects were observed in BL and DLBCL patients, respectively: neutropenia (80% versus 47.1%), anaemia (46.7% versus 5.9%), thrombocytopenia (53.3% versus 11.8%), bacterial pneumonia (33.3% versus 5.9%), and sepsis (20% versus 5.9%) (*p* < 0.05). In the BL group, 10 (66.7%) patients died from treatment-related or tumour-related causes, 5 (33.3%) achieved complete response, 1 achieved partial response (6.7%), and 7 developed progressive disease. The 1-year overall survival and progression-free survival rates were 33.3%. Of the DLBCL patients, 3 (17.6%) died from treatment-related causes, 14 (82.4%) achieved complete response, and 3 had progressive disease. The 1-year overall survival and progression-free survival rates were 82.4%. The strongest risk factor for death was relapse between chemotherapy cycles (adjusted hazard ratio = 47.3; 95%CI, 4.2–528.6, *p* = 0.002). Initiating antiretroviral therapy before chemotherapy failed to improve overall survival. DLBCL patients demonstrated good responses and survival outcomes, while BL patients could not tolerate chemotherapy due to more severe toxicity, and showed poor responses and survival outcomes.

## Introduction

Malignant lymphoma is a rapidly growing, aggressive, AIDS-defining cancer that results in death within several weeks or months if left untreated. Some studies have reported that the prevalence of lymphoma in HIV-infected patients has decreased dramatically with the introduction of antiretroviral therapy^1^. Despite convenient access to antiretroviral regimens through the National Free Antiretroviral Treatment Program (NFATP) in China^2^, HIV-related lymphomas—mainly Burkitt lymphoma (BL) and diffuse large B-cell lymphoma (DLBCL)—continue to lead to morbidity and mortality in Chinese HIV-infected patients due to delayed diagnosis of HIV infection, with lymphoma often being the first indicator of their disease^3^.

In the era of combination antiretroviral therapy (cART), CHOP-based regimens have been widely used to treat DLBCL in HIV-infected patients as they have better efficacy than other chemotherapy regimens^4^. Coutinho *et al*.^5^ demonstrated that HIV-infected patients diagnosed with DLBCL in the cART era have an excellent outcome when treated with CHOP-R, with an overall survival rate of 78%. CODOX-M/IVAC, a non-cross-resistant, intensive chemotherapy regimen developed by the American National Cancer Institute, confers high cure rates in HIV-infected patients with BL. With regard to toxicity, Rodrigo *et al*.^6^ reported that intensive chemotherapy with CODOX-M/IVAC ± R yielded an acceptable toxicity profile and good survival rates in patients with HIV-associated BL receiving cART. However, Mead *et al*.^7^, in the United Kingdom Lymphoma Group LY06 Study, found that toxicity was severe, especially in terms of myelosuppression and mucositis. However, the above conclusions are derived from Euro-American HIV-infected patients with lymphoma; the efficacy and tolerability of these chemotherapy regimens in Chinese HIV-infected patients remains obscure.

Beijing Ditan Hospital is an HIV/AIDS referral hospital in China. It provides care and treatment to HIV/AIDS patients, including those with lymphoma. The objective of this observational study was to evaluate the efficacy and tolerability of chemotherapy in Chinese HIV-infected patients with lymphoma.

## Methods

### Ethical considerations

This retrospective observational cohort study was approved by institutional review board of Beijing Ditan Hospital, the Capital Medical University, and complied with the principles of the Declaration of Helsinki. The clinical and therapeutic data were anonymously used and were abstracted from electronic medical records in Ditan Hospital.

### Subjects

We included all HIV-infected patients with newly diagnosed DLBCL treated with a CHOP regimen with or without rituximab (CHOP ± R) and newly diagnosed BL treated with a CODOX-M/IVAC regimen with or without rituximab (CODOX-M/IVAC ± R) at the Centers for Infectious Diseases, Beijing Ditan Hospital, the largest referral hospital for HIV/AIDS patients in North China, from January 2009 to August 2015. HIV-infected patients diagnosed with DLBCL or BL who did not receive chemotherapy were excluded. We investigated the efficacy, tolerability, and adverse effects of chemotherapy in these patients.

### Diagnosis and risk evaluation

Experienced pathologists used standard diagnostic criteria to diagnose DLBCL or BL on lymph node and bone marrow histopathological and immunohistochemical examination. The standard diagnostic criteria for BL included identification of C-MYC translocation by fluorescence *in situ* hybridization or of the t(8;14). Immunohistochemical analysis for BCL-2 and MYC expression was performed on paraffin-fixed tissue sections and BCL-2 and MYC immunostaining was carried out with anti-Bcl-2 antibody (abcam ab32124 1:250) and anti-myc antibody (abcam ab32072 1:200) using the BCL-2 and MYC expression diagnostic kit. The cut-offs for immunohistochemical markers for diagnosis of DLBCL were Bcl2 >70% and myc >40%, based on previous reports^8, 9^.

We used Han’s cell-of-origin algorithm^10^ to define germinal centre B-cell-like (GCB) or non-GCB DLBCL. Epstein-Barr virus (EBV) small ribonucleic acids were detected using *in situ* hybridization and an EBV *in situ* hybridization kit (ZSGB-Bio ISH502) with EBV-encoded small nuclear early region oligonucleotides on formalin-fixed paraffin-embedded tissue sections^11^. EBV-negative lymphoid tissues were treated as negative controls.

The Ann Arbor staging system was used to determine lymphoma stage. Staging investigations included physical examination; routine haematological and biochemical tests; lymph node mass measurement; bone marrow biopsy; and computerized tomography of the brain, chest, abdomen, and pelvis. Patients were regarded as high-risk based on following features: Ann Arbor stage III or IV; elevated lactate dehydrogenase levels; and tumour bulk >10 cm. Patients not meeting these criteria were regarded as low-risk^6, 7^.

Infectious complications during chemotherapy due to immunosuppression and severe myelosuppression were diagnosed and managed according to the *Guidelines for Prevention and Treatment of Opportunistic Infections in HIV-Infected Adults and Adolescents* recommended by the United States Centers for Disease Control and Prevention (CDC)^12^.

### Treatment and definitions

Patients diagnosed with DLBCL received a CHOP ± R regimen^13, 14^ on a 21-day schedule for a total of 8 cycles, and all patients received intrathecal methotrexate 12.5 mg per cycle for central nervous system prophylaxis. Patients with BL were treated with a CODOX-M/IVAC ± R regimen for 4 cycles and intrathecal prophylaxis was provided to patients at high risk of central nervous system involvement. The *Guidelines for the Use of Antiretroviral Agents in HIV-1-Infected Adults and Adolescents* of the United States CDC^15^ recommend cART for all HIV-infected patients to reduce the risk of disease progression; in patients with some conditions, such as AIDS-defining illnesses, more urgent initiation of therapy is favoured. In this study, patients with non-Hodgkin lymphoma, an AIDS-defining illness, were advised to initiate cART as soon as possible after being diagnosed with lymphoma, regardless of CD4 cell count. In this study, all patients were initiated on cART prior to receiving chemotherapy, based on the above-stated criteria. The preferred regimen was tenofovir, lamivudine, plus efavirenz, as recommended by the Chinese *National Free HIV Antiretroviral Treatment Handbook* ^16^. In this study, patients who did not start cART until diagnosis of lymphoma initiated treatment with tenofovir, lamivudine, plus efavirenz, while those who had been receiving cART regimens such as zidovudine, lamivudine, plus nevirapine or efavirenz, were switched to tenofovir, lamivudine, plus efavirenz prior to chemotherapy to reduce the risk of zidovudine-associated myelosuppression and nevirapine-induced liver injury when combined with chemotherapy.

According to the *Guidelines for Prevention and Treatment of Infectious Complications in HIV-Infected Adults and Adolescents* recommended by the United States CDC^12^, HIV-infected adults and adolescents with a CD4 count <200 cells/µL should receive chemoprophylaxis against pneumocystis pneumonia. Trimethoprim-sulfamethoxazole (TMP-SMX) is the recommended prophylactic agent, and 1 double-strength tablet daily is the preferred regimen. Patients with a CD4 count <50 cells/µL should receive chemoprophylaxis against disseminated *Mycobacterium avium* complex disease; azithromycin 1200 mg once weekly is the preferred prophylactic agent. In this study, because the cART regimen used was tenofovir, lamivudine, plus efavirenz, primary prophylaxis against hepatitis B virus infection was not needed, as tenofovir and lamivudine have antiviral activity against hepatitis B virus^15^. All study patients received primary prophylaxis against pneumocystis pneumonia and *Mycobacterium avium* complex. Patients received granulocyte-colony-stimulating factor as primary prevention due to severe bone marrow suppression after receiving chemotherapy.

Patients not on cART when diagnosed with lymphoma received an antiretroviral regimen of tenofovir, lamivudine, plus efavirenz, prior to initiation of chemotherapy. Timely laboratory monitoring of HIV viral load was performed based on the Chinese *National Free HIV Antiretroviral Treatment Handbook* ^16^, and the antiretroviral effect was evaluated in patients receiving chemotherapy.

Complete response to chemotherapy was defined as complete disappearance of disease as determined by all available clinical, biochemical, and radiological evidence. Partial response required at least a 50% decrease in the sum of the product of the greatest diameters. Progressive disease (non-response) required assessable disease progression or the appearance of any new lesion(s) during or at the end of chemotherapy^17^. Relapse in the interval between chemotherapeutic cycles was defined as disease reduction or even complete disappearance during chemotherapy, but relapse or appearance of primary or new lesions in the interval between chemotherapeutic cycles, based on clinical and radiological evidence.

For patients who survived until discharge, the date last known alive was obtained from the record of the last follow-up in the database in CDC in China. The major end-points in this study included overall survival, defined as the time from initial diagnosis to death, and progression-free survival (PFS), defined as the time from the first day of treatment to the date at which lymphoma progressed or the date on which the patient died from any cause^18^.

### Statistical analysis

All statistical data were analysed using SPSS software, version 19.0 (SPSS Institute, Chicago IL, USA). Continuous variables with a skewed distribution are presented as the median with the minimum and maximum range. Categorical variables are presented as numbers and percentages; chi-square or Fisher’s exact tests were used to compare patient characteristics, as appropriate. Column charts were used to illustrate grade 3–4 adverse effects following chemotherapy in the DLBCL and BL group. The log-rank test was used to compare survival distributions of overall survival and PFS between the DLBCL and BL groups. Cox proportional hazards regression models were used to evaluate the association between the following variables and death among HIV-infected patients with lymphoma receiving chemotherapy: baseline CD4 count, initiation of cART prior to chemotherapy, receiving rituximab during therapy, and relapse in the interval between chemotherapeutic cycles. A significance level of 0.05 was set for all statistical testing.

## Results

### Patient characteristics

From January 2009 to August 2015, 17 patients were diagnosed with DLBCL and received chemotherapy with a CHOP ± R regimen, while 15 patients were diagnosed with BL and were treated with a CODOX-M/IVAC ± R regimen. In the DLBCL group, 6 patients (35.3%) were on cART at baseline, 11 (64.7%) initiated cART prior to chemotherapy, and 13 (76.5%) received rituximab in addition to CHOP regimen. In the BL group, 4 patients (26.7%) were on cART at baseline, 11 (73.3%) started cART prior to receiving chemotherapy, and 11 (73.3%) received rituximab during chemotherapy (CODOX-M/IVAC + R). The demographic and clinical features of these patients are detailed in Table 1.

**Table 1:** Clinical characteristics of HIV-infected patients with lymphoma receiving chemotherapy.

Characteristic	DLBCL (*n* = 17)	Burkitt lymphoma (*n* = 15)
**Age (mean ± SD)**	42.6 ± 13.3	39.8 ± 8.5
<50 years (%)	11 (64.7)	13 (86.7)
≥50 years (%)	6 (35.3)	2 (13.3)
**Male sex**, ***n*** **(%)**	16 (94.1)	15 (100.0)
**Transmission route**, ***n*** **(%)**		
Homosexual sexual	7 (41.2)	8 (53.3)
Heterosexual sexual	8 (47.1)	7 (46.7)
Transfusion	2 (11.8)	0 (0.0)
**HIV-related characteristics**		
Baseline viral load, copies/mL [median (range)]	9039 (0–284331)	39294.5 (0–849556)
Baseline CD4 count, cells/µL [median (range)]	104 (3–747)	266.5 (11–415)
CD4 count < 50 cells/µL, *n* (%)	4 (23.5)	1 (6.7)
CD4 count 50–199 cells/µL, *n* (%)	5 (29.4)	6 (40.0)
CD4 count ≥ 200 cells/µL, *n* (%)	8 (47.1)	8 (53.3)
Having received cART at baseline (%)	6 (35.3)	4 (26.7)
cART initiation prior to chemotherapy (%)	11 (64.7)	11 (73.3)
**Ann Arbor stage**, *n* (%)		
I	0 (0.0)	0 (0.0)
II	5 (29.4)	5 (33.3)
III	7 (41.2)	2 (13.3)
IV	5 (29.4)	8 (53.4)
**Karnofsky performance status**, *n* (%)		
<40%	7 (41.2)	11 (73.3)
40–60%	6 (35.3)	3 (20.0)
>70%	4 (23.5)	1 (6.7)
**International prognostic index score**, *n* (%)		
0	0 (0.0)	0 (0.0)
1	3 (17.6)	1 (6.7)
2	8 (47.1)	5 (33.2)
3	6 (35.3)	8 (53.4)
4	0 (0.0)	1 (6.7)
**LDH**, U/L [median (range)]	250.4 (152.1–937.9)	364.8 (209.1–2664.9)
LDH > upper limit of normal (%)	10 (58.8)	14 (93.3)
**B symptoms**, *n* (%)	3 (17.6)	6 (40.0)
**Bulky tumour (>10 cm**), *n* (%)	5 (29.4)	9 (60.0)
**Rituximab-containing therapy**, *n* (%)	13 (76.5)	11 (73.3)

HIV, human immunodeficiency virus; DLBCL, diffuse large B-cell lymphoma; SD, standard deviation; cART, combination antiretroviral therapy; LDH, lactate dehydrogenase.

### Pathological features of study participants

The pathological diagnosis was determined by 2 separate pathologists in Ditan Hospital using immunohistochemical criteria. The cell of origin in the DLBCL arm was subdivided into GCB (10 cases) and non-GCB (7 cases) subtypes; 23.5% (4 cases) expressed *BCL-2*. BL was subdivided into EBV-positive (9 cases) and EBV-negative (6 cases) types. Fluorescence *in situ* hybridization was performed to identify c-MYC translocation in BL.

### Adverse events

**Figure 1:**
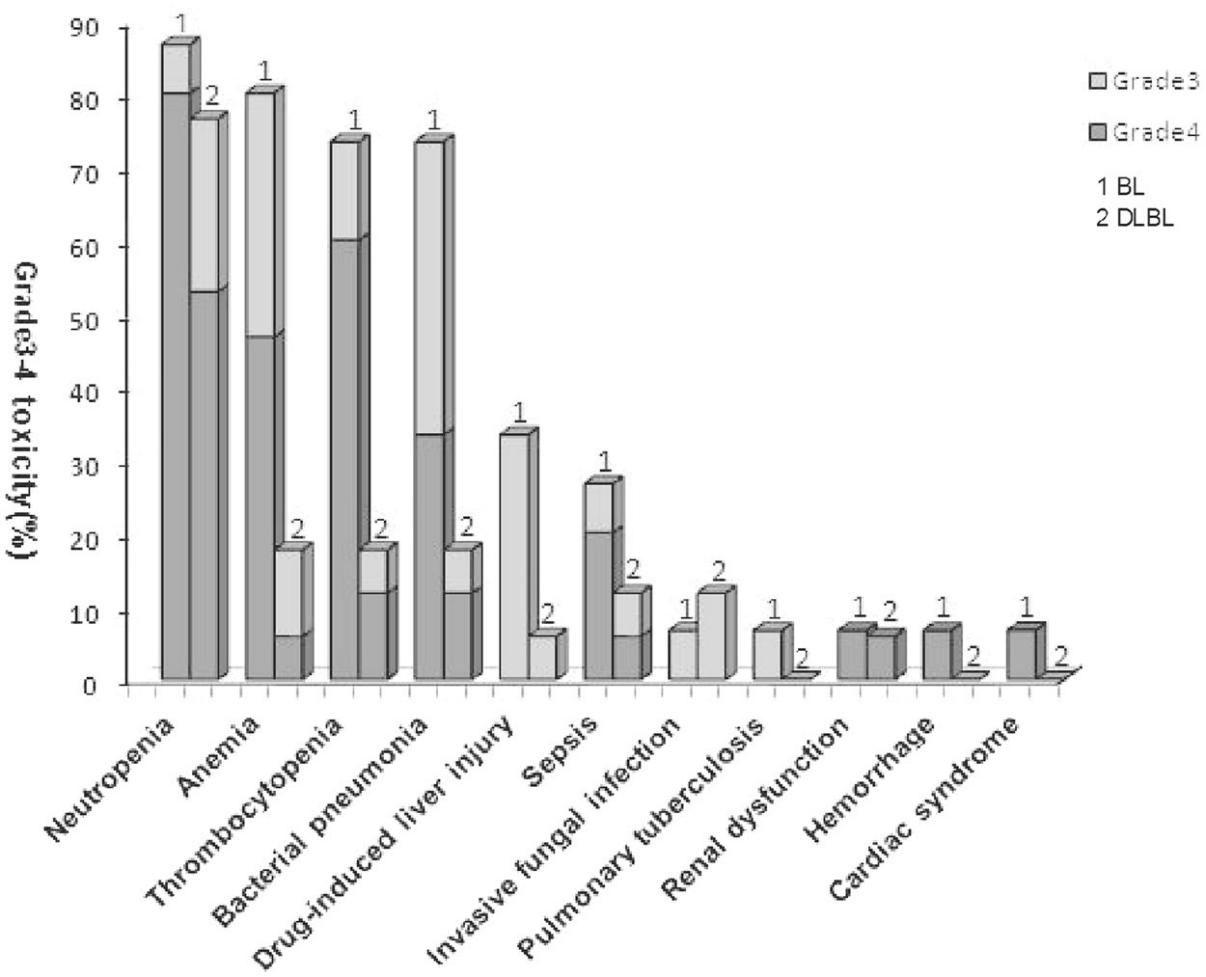
Grade 3/4 adverse effects in BL patients receiving CODOX-M/IVAC ± R and DLBCL patients receiving CHOP ± R chemotherapy.

The National Cancer Institute grade 3–4 toxicities observed during chemotherapy are summarised in Fig. 1. The proportion of patients in the BL and DLBCL arms, respectively, who experienced grade 4 adverse events were: neutropenia, 80% versus 47.1%; anaemia, 46.7% versus 5.9%; and thrombocytopenia, 53.3% versus 11.8% (*p* < 0.05). Patients also experienced grade 3–4 infectious complications, with 33.3% versus 5.9% in BL and DLBCL arms, respectively, developing bacterial pneumonia; 20% versus 5.9% developing sepsis; 6.7% versus 11.8% developing invasive fungal infections; and 6.7% versus 0.0% developing pulmonary tuberculosis. During the entire chemotherapy protocol, 8 BL patients (53.3%) required red blood cell transfusion (mean: 11 units), and 8 BL patients (53.3%) required transfusion of platelets (mean: 2.5 units) due to treatment-related myelosuppression. Only 1 DLBCL patient (5.9%) required red blood cell transfusion (of only 2 units).

A significantly greater proportion of patients in the BL group died of treatment related causes (*p* < 0.01). In the BL group, 7 (46.6%) patients died of treatment-related causes (septic shock [*n* = 4], haemorrhage [*n* = 1], severe arrhythmia [*n* = 1], and renal failure [*n* = 1]), while in DLBCL group, only 3 patients (17.6%) died of treatment-related causes (septic shock [*n* = 2] and renal failure [*n* = 1]) (Table 2). In terms of duration of chemotherapy, the mean cycle length in the BL group receiving the CODOX-M/IVAC ± R regimen was 28.5 days, significantly longer than the 20.9 days that the DLBCL group received the CHOP ± R regimen (*p* < 0.001).

**Table 2:** Outcome and causes of death following chemotherapy in HIV-infected patients with lymphoma.

Characteristic	DLBCL, n (%) (*n* = 17)	Burkitt lymphoma, n (%) (*n* = 15)
**Complete response**	**14 (82.4)**	**5 (33.3)**
**Partial response**	**0 (0.0)**	**1 (6.7)**
Death	0 (0.0)	1 (6.7)
Cause of death		
Tumour metastasis	0 (0.0)	1 (6.7)
**Progressive Disease**	**3 (17.6)**	**7 (46.6)**
Death	3 (17.6)	7 (46.6)
Cause of death		
Septic shock	2 (11.8)	2 (13.3)
Tumour metastasis	0 (0.0)	2 (13.3)
Renal failure	1 (5.8)	1 (6.7)
Haemorrhage	0 (0.0)	1 (6.7)
Severe arrhythmia	0 (0.0)	1 (6.7)
**Unable to evaluate**	**0 (0.0)**	**2 (13.3)**
Death	0 (0.0)	2 (13.3)
Cause of death		
Septic shock	0 (0.0)	2 (13.3)

HIV, human immunodeficiency virus; DLBCL: diffuse large B-cell lymphoma.

In the current observational cohort, patients receiving a CODOX-M/IVAC ± R regimen suffered from severe myelosuppression, with 8 patients (53.3%) requiring substantial volumes of red blood cells transfused and 8 (53.3%) requiring platelet transfusion during chemotherapy. Grade 3–4 infectious complications were the other main group of severe adverse events. Haemorrhage, arrhythmia, and renal failure were also fatal adverse events; these occurred in 3 patients during chemotherapy. The mean chemotherapy cycle length in the BL group receiving a CODOX-M/IVAC ± R regimen was significantly prolonged due to management of the above severe adverse events. Chemotherapy-associated deaths occurred in 7 patients (46.6%). These deaths were from septic shock, haemorrhage, arrhythmia, and renal failure.

Patients with DLBCL receiving CHOP ± R also experienced severe myelosuppression, mainly grade 3–4 neutropenia (76.5%); but only 1 patient required red blood cell transfusion during chemotherapy. Grade 3–4 infectious complications experienced by these patients included bacterial pneumonia, sepsis, and invasive fungal infection. There were 3 chemotherapy-associated deaths in this group (17.6%).

### Treatment response and survival outcomes

**Figure 2:**
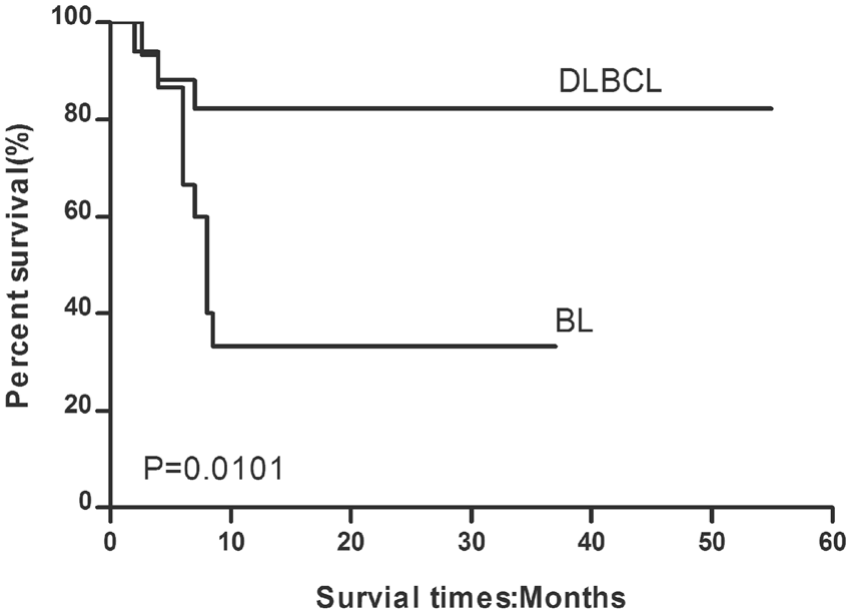
Overall survival curve for BL patients receiving CODOX-M/IVAC ± R and DLBCL patients receiving CHOP ± R chemotherapy.

**Figure 3:**
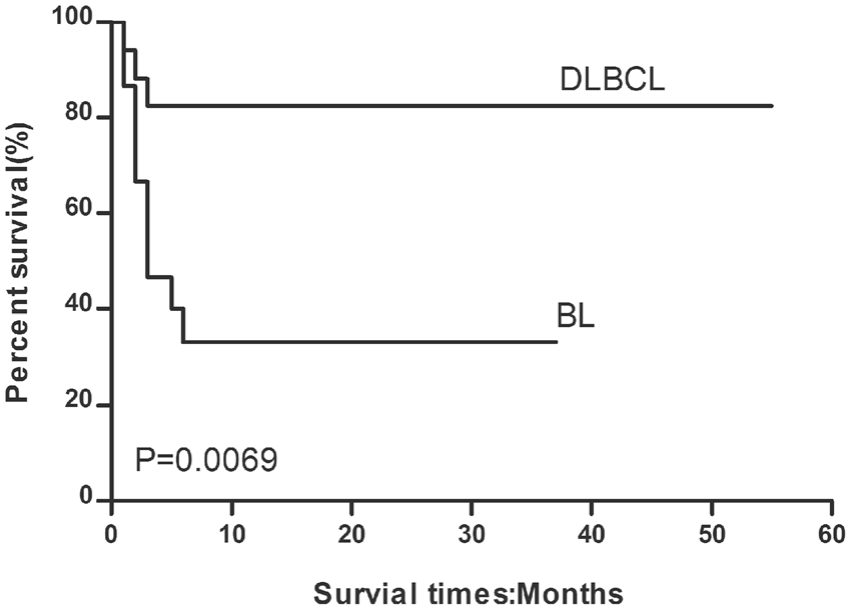
Progression-free survival curve for BL patients receiving CODOX-M/IVAC ± R and DLBCL patients receiving CHOP ± R chemotherapy.

The response to treatment, survival outcomes, and cause of death following chemotherapy in HIV-infected patients with lymphoma are listed in Table 2. Of the 17 patients with DLBCL receiving a CHOP ± R regimen, 14 (82.4%) achieved complete response, while 3 experienced progressive disease resulting in death (2 due to septic shock and 1 due to renal failure). Radiotherapy was initiated in 1 patient with BL who showed a poor response to 4 cycles of CODOX-M/IVAC; however, the response to radiotherapy was also poor. One-year overall survival was 82.4%, and the median overall survival duration was 26.6 months (range, 18.8–35.2 months). The overall mortality rate in this group was 17.6%. The 1-year PFS rate was 82.4%, with a median PFS of 25.8 months (range, 17.8–34.1 months), as shown in Figs 2 and 3.

Of the 15 patients with BL receiving CODOX-M/IVAC ± R regimen, 5 (33.3%) achieved complete response, 1 achieved partial response (6.7%) and died within 2 months of completing chemotherapy due to progressive disease, while the other 7 patients experienced progressive disease and died during chemotherapy (septic shock [*n* = 4], haemorrhage [*n* = 1], severe arrhythmia [*n* = 1], and renal failure [*n* = 1]). Two other patients (13.3%) died of septic shock early during the course of chemotherapy, before treatment response could be evaluated. The overall mortality rate in this group was 66.7%. One-year overall survival was 33.3%, and the median overall survival duration was 13.8 months (range, 8.4–20.3 months). One-year PFS was 33.3%, and median PFS was 11.4 months (range, 5.4–18.5 months), as shown in Figs 2 and 3.

### Relapse in the interval between chemotherapy cycles

Of the 17 patients with DLBCL who received the CHOP ± R regimen, 14 attained complete response while 3 had progressive disease with relapse in the interval between chemotherapy cycles: One relapsed between the first and second cycle, 1 between the fourth and fifth cycle, and 1 between the sixth and seventh cycle. All 3 patients died. Of the 15 patients with BL who received the CODOX-M/IVAC ± R regimen, 7 progressed, with relapse between the first and second cycle resulting in poor prognosis.

In the multivariate analysis, the strongest risk factor for death among HIV-infected patients with lymphoma receiving chemotherapy was relapse in the interval between chemotherapy cycles (adjusted hazard ratio [AHR], 47.3; 95% CI, 4.2–528.6; *p* = 0.002). Baseline CD4 count, initiation of cART prior to chemotherapy, and receiving rituximab during therapy were not statistically significantly associated with prognosis.

### Evaluation of HIV viral load after completion of chemotherapy

Of the 32 patients receiving chemotherapy, 22 completed it (including 14 DLBCL patients receiving CHOP ± R and 8 BL patients receiving CODOX-M/IVAC ± R). HIV viral load was suppressed below the limit of detection of commercially available assays after completion of chemotherapy.

## Discussion

This retrospective study was the first conducted in the Chinese mainland to evaluate the tolerability, efficacy, and outcome of chemotherapy in Chinese HIV-infected patients with AIDS-related BL and DLBCL. In the era of cART, standard dose chemotherapeutic regimens for DLBCL^4^ and intensive regimens for BL^6^ are recommended and have been used with success. In this study, we found that patients with DLBCL receiving standard dose CHOP ± R chemotherapy experienced severe adverse events, but that the regimen yielded good efficacy and survival outcomes. In patients with BL, however, intensive chemotherapy with CODOX-M/IVAC ± R was not feasible; it was poorly tolerated and yielded poor survival rates, being unable to overcome BL in our cohort. An important finding in this study was that relapse in the interval between chemotherapy cycles was the strongest risk factor for death in HIV-infected patients with lymphoma receiving chemotherapy. This was demonstrated for patients with BL and DLBCL, indicating a trend toward poor prognosis during chemotherapy despite the small number of patients. In this study, 11 patients experienced relapse in the interval between chemotherapy cycles; the mechanisms underlying relapse may be an area worthy of further elucidation in Chinese HIV-infected patients with lymphoma.

Although the NFATP provides free antiretroviral medications to HIV-infected patients in China, the treatment of complications and co-morbidities, including AIDS-defining diseases such as lymphoma, must be paid for by the patients themselves^18^. Hence, 7 patients did not receive rituximab due to cost constraints. It has been reported that rituximab (a CD20-directed monoclonal antibody) improves overall survival and combines safely with the standard dose chemotherapeutic regimens, CHOP (for DLBCL)^19^ and CODOX-M/IVAC (for BL)^20^ in HIV-infected patients. In contrast, Kaplan *et al*.^21^ demonstrated in the AIDS-malignancies consortium trial 010, that rituximab did not improve clinical outcomes in a randomized phase 3 trial of CHOP ± R in patients with HIV-associated non-Hodgkin lymphoma. Wasterlid *et al*.^22^ also reported that rituximab was not significantly associated with improved overall survival in HIV-infected patients with BL treated with intensive regimens. In the present study, the multivariate analysis indicated that receiving rituximab during chemotherapy was not statistically significantly associated with prognosis. No adverse reactions, apart from 1 case of hypersensitivity reaction, were observed in the 24 patients who received rituximab, indicating that rituximab can be combined safely with the chemotherapeutic regimens used. However, use of rituximab did not show a trend favouring improvement in overall survival in our cohort.

Recent studies^6^ demonstrated that antiretroviral regimen can be combined safely with intensive CODOX-M/IVAC or standard dose CHOP chemotherapy. In this study, most patients (22/32 cases) were not receiving cART as they were unaware of their HIV status until lymphoma—the first indicator of their disease—was diagnosed. These patients initiated cART prior to chemotherapy, whereas the other 10 patients were already taking cART when diagnosed with lymphoma. Of 32 the patients receiving chemotherapy, 22 completed it, and none had a detectable HIV viral load after completion of chemotherapy, indicating that the cART regimen used (tenofovir, lamivudine, plus efavirenz) did not interact with the chemotherapeutic regimens, and can combined safely with intensive CODOX-M/IVAC ± R or standard dose CHOP ± R chemotherapy in Chinese HIV-infected patients.

It is undisputable that long-term concomitant cART improves overall survival in patients with AIDS-related BL or DLBCL receiving chemotherapy. Chiang *et al*.^23^ demonstrated that short-term cART did not improve immune function, especially in severely immunosuppressed patients. In this study, 22 patients did not initiate cART until immediately prior to receiving chemotherapy. The multivariate analysis indicated that initiating cART prior to chemotherapy was not statistically significantly associated with prognosis, implying that initiating cART prior to receiving chemotherapy does not improve overall survival in patients with AIDS-related BL or DLBCL.

In this study, we found that intensive chemotherapy with CODOX-M/IVAC ± R yielded severe toxicity because of lower Karnofsky performance status and lower CD4 cell counts. In Chinese patients with HIV-associated lymphoma, most patients were not diagnosed with HIV infection until lymphoma became the first indicator of their disease. Hence, they presented with late-stage disease (AIDS) and lower CD4 counts, making them prone to infectious complications, resulting in lower Karnofsky performance status and intolerance of chemotherapy-induced toxicities. In this study, the intensive chemotherapeutic regimen, CODOX-M/IVAC ± R, was used to treat BL in HIV-infected patients in China, but these patients had poor clinical response rates, poor survival outcomes, and could not tolerate chemotherapy due to severe toxicity. The AMC 048 study^24^ indicated that, using modified CODOX-M/IVAC-R regimen, the 1-year overall survival was 72% among patients with a Karnofsky performance status >30. The Northern Italy Leukemia Group adopted the B-NHL 2002 regimen to treat a cohort of 105 consecutive, unselected adult patients with BL and demonstrated on multivariate analysis that patients with an Eastern Cooperative Oncology Group performance status >1 did very poorly^25^. In this study, Karnofsky performance status was <40% in 11 patients (73.3%) in the BL group, and intensive chemotherapy with CODOX-M/IVAC ± R was associated with severe toxicity and poor survival rates, indicating that patients with poor performance status also do poorly in China, and suggesting that intensive chemotherapy is not recommended for patients with lower Karnofsky performance status scores. Dunleavy *et al*.^26^ reported low-intensity treatment consisting of infused etoposide, doxorubicin, and cyclophosphamide with vincristine, prednisone, and rituximab (EPOCH-R) for patients with untreated BL. Their results indicated that a lower-dose short-course combination with a double dose of rituximab (SC-EPOCH-RR)-based treatment was highly effective in adults with sporadic or immunodeficiency-associated BL. Low-intensity EPOCH-R-based treatment may be a good chemotherapeutic option for HIV-infected patients with BL in China.

In summary, HIV-infected patients with BL and DLBCL receiving chemotherapy experienced severe myelosuppression and infectious complications due to the overall poor performance status and relatively low CD4 counts. Patients with DLBCL demonstrated good clinical responses and survival outcomes when treated with CHOP ± R, but patients with BL could not tolerate chemotherapy with CODOX-M/IVAC ± R due to more severe toxicity; and their clinical response and survival outcomes were poor due to poor performance status and lower CD4 counts. Intensive chemotherapy with CODOX-M/IVAC ± R is not recommended for patients with BL with lower Karnofsky performance status scores and lower CD4 counts in China. Relapse in the interval between chemotherapeutic cycles was the strongest risk factor for death among patients with lymphoma receiving chemotherapy. Short-term cART prior to chemotherapy failed to improve overall survival, but long-term concomitant cART is important to prevent lymphoma and enable successful chemotherapy to treat lymphoma.
